# The ^1^H NMR serum metabolomics response to a two meal challenge: a cross-over dietary intervention study in healthy human volunteers

**DOI:** 10.1186/s12937-019-0446-2

**Published:** 2019-04-08

**Authors:** Millie Rådjursöga, Helen M. Lindqvist, Anders Pedersen, Göran B. Karlsson, Daniel Malmodin, Carl Brunius, Lars Ellegård, Anna Winkvist

**Affiliations:** 1 Department of Internal Medicine and Clinical Nutrition, Sahlgrenska Academy, University of Gothenburg, Gothenburg, Sweden; 2 Swedish NMR Centre, University of Gothenburg, Gothenburg, Sweden; 3 Department of Biology and Biological Engineering Food and Nutrition Science Chalmers University of Technology, Gothenburg, Sweden

**Keywords:** Metabolomics, Nutrition, NMR, OPLS-DA, OPLS-EP, ANOVA-PLS, Postprandial, Serum, Breakfast

## Abstract

**Background:**

Metabolomics represents a powerful tool for exploring modulation of the human metabolome in response to food intake. However, the choice of multivariate statistical approach is not always evident, especially for complex experimental designs with repeated measurements per individual. Here we have investigated the serum metabolic responses to two breakfast meals: an egg and ham based breakfast and a cereal based breakfast using three different multivariate approaches based on the Projections to Latent Structures framework.

**Methods:**

In a cross over design, 24 healthy volunteers ate the egg and ham breakfast and cereal breakfast on four occasions each. Postprandial serum samples were subjected to metabolite profiling using ^1^H nuclear magnetic resonance spectroscopy and metabolites were identified using 2D nuclear magnetic resonance spectroscopy. Metabolic profiles were analyzed using Orthogonal Projections to Latent Structures with Discriminant Analysis and Effect Projections and ANOVA-decomposed Projections to Latent Structures.

**Results:**

The Orthogonal Projections to Latent Structures with Discriminant Analysis model correctly classified 92 and 90% of the samples from the cereal breakfast and egg and ham breakfast, respectively, but confounded dietary effects with inter-personal variability. Orthogonal Projections to Latent Structures with Effect Projections removed inter-personal variability and performed perfect classification between breakfasts, however at the expense of comparing means of respective breakfasts instead of all samples. ANOVA-decomposed Projections to Latent Structures managed to remove inter-personal variability and predicted 99% of all individual samples correctly. Proline, tyrosine, and N-acetylated amino acids were found in higher concentration after consumption of the cereal breakfast while creatine, methanol, and isoleucine were found in higher concentration after the egg and ham breakfast.

**Conclusions:**

Our results demonstrate that the choice of statistical method will influence the results and adequate methods need to be employed to manage sample dependency and repeated measurements in cross-over studies. In addition, ^1^H nuclear magnetic resonance serum metabolomics could reproducibly characterize postprandial metabolic profiles and identify discriminatory metabolites largely reflecting dietary composition.

**Trial registration:**

Registered with ClinicalTrials.gov, identifier: NCT02039596. Date of registration: January 17, 2014.

**Electronic supplementary material:**

The online version of this article (10.1186/s12937-019-0446-2) contains supplementary material, which is available to authorized users.

## Background

To establish associations and causation between diet and health, objective and reliable methods are needed to measure dietary exposure [1]. Unfortunately, few such methods exist. Instead, subjective assessment methods are commonly used and these include dietary records, food diaries, 24-h dietary recalls, food frequency questionnaires and diet history records. These methods rely on subjects’ own reports of their diets [2]. As such, they are associated with difficulties in estimation of consumption over time and of portion size, variation in dietary intake, cognitive processes such as episodic and generic memory as well as bias in over- and under-reporting of foods [2]. Despite validation efforts, random and systematic errors in subjective dietary assessment methods limit the possibility to measure true dietary intake [3]. A few single dietary biomarkers are today used to validate or to substitute subjective dietary assessment methods [1, 4–6]. Still, currently used dietary biomarkers do not always correlate well with the nutrients or foods they are intended to indicate and they fail to reflect the complex matrix of an overall diet [3]. Providing accurate and reliable measurements of dietary exposure constitutes one of the most challenging problems in nutrition research today [7].

Metabolomics concentrates on the high-throughput characterization of small molecule metabolites (< 1.5 kDa) in biological samples and is therefore a method suitable to explore metabolic effects of dietary exposures [8]. Using metabolomics, food-derived metabolites and the change in endogenous metabolites can be identified including amino acids, alkaloids, polyphenols and metabolites of microbial origin. Metabolomics in nutrition has been described as: “The study of endogenous and gut microbiota metabolic response to food (general diet or intervention) and the identification of metabolites that originate from food and could be used as biomarkers of exposure of these foods” [9]. In the area of nutritional metabolomics, potential biomarkers for individual food items and diets have been identified in both urine and plasma/serum [10]. However, further studies are needed to deepen the understanding of the food metabolome and to identify additional potential nutritional biomarkers for food items and complex diets. Controlled dietary intervention studies, where true consumption can be monitored in a supervised fashion, provide an opportunity to investigate the metabolic response of different foods or diets using metabolomics. In clinical studies that use metabolomics to explore the response to different exposures, it is not self-explanatory what method to apply regarding data analysis. Multivariate methods based on projections to latent structures (PLS) with different extensions are frequently applied, and orthogonal projections to latent structures with discriminant analysis (OPLS-DA) has become a standard method in metabolomics [11–13]. These multivariate methods are in their standard form suitable only for independent data, as they do not by themselves separate within- from between-individual effects but rather the average effect between two or more sample classes [14]. However, clinical studies may entail sample dependency from repeated measures on the same investigated unit. Using an independent test on dependent data generates less robust models and potentially both false positive and negative discriminatory metabolites. OPLS-EP is an extension of the OPLS-DA method developed to handle dependent data by data pre-processing [15]. Using OPLS-EP the variation within and between subjects is separated and intrinsic differences in treatment effects between individuals can be identified [15]. However, a drawback lies in that only one measurement per treatment can be used and, consequently, averages must be used from repeated measurements on the same individual instead of concurrently examining all samples. ANOVA decompositioning of multivariate data offers a means to subtract study factors from measured variables, thus providing the possibility to focus on the reproducible within-person effect (i.e. treatment) while still keeping all individual samples in the analysis [16, 17].

The aim of the present study was to explore the outcome, discriminative potential, reproducibility of metabolic profiles, and biological relevance of discriminatory metabolites, of three different multivariate models, OPLS-DA, OPLS-Effect Projections (EP), and ANOVA-PLS. This was performed in a nutritional cross-over intervention study with the aim to investigate the serum metabolic response to two isocaloric breakfast meals using ^1^H NMR metabolomics. We have previously described the urine metabolome of the same diet [18].

## Materials and methods

### Ethical approval, recruitment and subject screening

The project was approved by the Regional Ethical Review Board in Gothenburg, Sweden (reference number 561–12), adhered to the Helsinki Declaration, and registered with ClinicalTrials.gov (identifier: NCT02039596).

Volunteers were recruited by advertisement at the University of Gothenburg, Sweden, and Chalmers University of Technology in Gothenburg, Sweden. Before entering the study participants provided written informed consent.

In total, 24 healthy volunteers, 12 males and 12 females, were enrolled in the study (Table 1). Volunteers were considered suitable if apparently healthy (normal serum electrolytes, iron status, creatinine, liver transaminases, bilirubin and alkaline phosphatase, C-reactive protein, plasma glucose, and thyroid status), with no regular use of medications (contraceptives were permitted), and BMI > 18.5 and < 30 kg/m^2^. Screening included a three-day weighed-food diary a short lifestyle questionnaire that included questions regarding food and alcohol consumption, use of nicotine, drugs, herbal remedies and supplements and level of physical activity. Body composition was measured with bioimpedance (ImpediMed Bioimp Version 5.3.1.1). Exclusion criteria included: aged <18 or >65 years, pregnancy or lactation, use of nicotine, natural remedies and/or herbal tea, alcohol consumption higher than 5 units per week (1 unit = 12 g alcohol), allergies to food items included in the study, and the practice of an extreme diet or intent to change physical activity and/or dietary habits before or during the intervention.

**Table 1:** Anthropometric characteristics of included volunteers (*n* = 24)

Characteristics	Males (*n* = 12)		Females (*n* = 12)	
	mean ± SD	min/max	mean ± SD	min/max
Age (year)	27.3 ± 11.2	19.0/54.0	24.4 ± 8.2	18.0/46.0
Height (cm)	184.3 ± 6.0	172.0/192.0	169.2 ± 6.1	159.0/177.0
Body weight (kg)	77.5 ± 7.8	66.8/91.3	66.2 ± 7.1	56.6/77.4
BMI (kg/m2)	22.8 ± 2.1	20.6/26.9	23.1 ± 2.3	19.5/26.7
Fat mass (%)	13.9 ± 5.9	6.7/24.7	26.9 ± 5.2	18.4/34.0

### Study design

Study participants consumed the two different breakfasts, cereal breakfast (CB) and egg and ham breakfast (EHB), four times each, in the following order; abba/baab, where a = CB and b = EHB, Tuesday to Friday during two consecutive weeks (eight occasions in total). Breakfasts were consumed at the Department of Internal Medicine and Clinical Nutrition, University of Gothenburg, Sweden. The CB consisted of orange juice, oat puffs with milk, and a rye bread sandwich with hard cheese and fresh tomato. The EHB consisted of orange juice, scrambled eggs, white beans in tomato sauce, fried pork loin, tomato and toasted white bread with orange marmalade. Study participants choose either coffee (male *n* = 6, female *n* = 4) or tea (male *n* = 6, female *n* = 8), both with 20 ml of milk. Participants were also given a choice between a large (750 kcal) (male *n* = 12, female *n* = 1) or a small (500 kcal) (female *n* = 11) breakfast size.

The two breakfast meals had similar composition of protein, fat and carbohydrates. Breakfasts of 500 kcal comprised 20 g protein, 19 g fat and 60 g carbohydrates while the breakfasts of 750 kcal comprised 29 g protein, 34 g fat and 80 g carbohydrates (see supplementary file information of previous article [18] for detailed meal composition and Additional files 1, 2 and 3 for detailed description of breakfasts’ nutrients, amino acids and fatty acids).

Two weeks before and during the intervention, study participants were asked to refrain from using dietary supplements and occasional medications. The day before and during the intervention, volunteers were asked to abstain from drinking alcohol, engaging in strenuous exercise (> 2 h moderate intense physical activity, defined as 3–6 MET:s (metabolic equivalents)) [19], and eating fish. Volunteers did not have any other restrictions regarding food consumption.

**Fig. 1:**
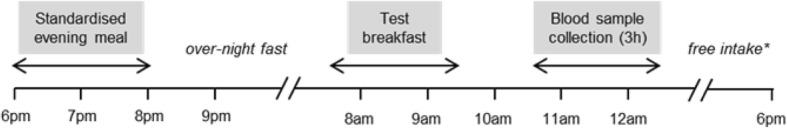
Study design of clinical intervention, Monday evening to Friday lunch during two consecutive weeks. *Volunteers were instructed to abstain from eating fish, dietary supplements and drinking alcohol during the intervention

To help stabilize background metabolic profiles further, a standardized evening meal of quenelles with tagliatelle in tomato sauce (488 kcal) was provided to be consumed between 18:00 and 20:00 h (Fig. 1). Volunteers were instructed to drink water for the evening meal, not eat anything further and only drink water before arriving to the test kitchen between 07.30 and 09.30 h where they consumed the breakfasts within 30 min. During the intervention, volunteers noted health status, occasional medications, and exact time of evening meal together with water intake during the overnight fast.

### Serum collection and preprocessing

Postprandial serum samples were collected after breakfast meals (3 h 15 min ± 22 min). In total, 192 samples were collected. Venous blood was drawn into 4 mL Z serum Separator Clot activator tubes (VACUETTE® TUBE Greiner Bio-One), allowed to clot at 4 °C for 30 min and centrifuged at 4 °C at 2600 x g for 10 min. 400 μL serum was aliquoted in 500 μL cryo vials and placed at − 20 °C within 1 h (57 min ± 11 min) and at − 80 °C within 2 h. Samples were stored at − 80 °C until analysis. ^1^H-nuclear magnetic resonance (NMR) spectroscopy analysis was performed on all serum samples. Prior to ^1^H-NMR analysis, serum samples were thawed for 60 min at 4 °C, 100 μL serum was mixed with 100 μL phosphate buffer (75 mM Na_2_HPO_4_, 20% D_2_O, 0.2 mM imidazole, 4% NaN_3_, 0.08% TSP-d_4_, pH 7.4) in a deep well plate. 180 μL sample mix was transferred to 3.0 mm NMR tubes (Bruker BioSpin, 96 sample racks for SampleJet) using a SamplePro liquid handling robot (Bruker BioSpin, Rheinstetten, Germany). Samples were kept at 6 °C until analysis. For quality control three samples with pooled serum from four individuals in the dataset and three buffer samples were used on each 96 sample rack.

### NMR spectroscopy analysis

All ^1^H NMR spectra were measured on an Oxford 800 MHz magnet equipped with a Bruker Avance III HD console and with a 3 mm TCI cryoprobe and a cooled (6 °C) SampleJet automatic sample changer for sample handling. All ^1^H NMR experiments were performed at 298 K. NMR data (1D perfect echo with excitation sculpting for water suppression) was recorded using the Bruker pulse sequence ‘zgespe’. The spectral width was 20 ppm, the relaxation delay was 1.34 s. The acquisition time was 2.04 s. With a total of 64 scans collected into 64 k data points, the measurement time for each sample was 4 min 19 s. All data sets were zero filled to 128 k and an exponential line-broadening of 0.3 Hz was applied before Fourier transformation. All data processing was performed with TopSpin 3.2pl6 (Bruker BioSpin, Rheinstetten, Germany). TSP-d_4_ was used for referencing. ^1^H NMR data were acquired for a total of 192 serum samples.

For annotation, pooled serum from all individuals in the dataset was utilized for natural abundance ^1^H-^13^C HSQC (‘hsqcetgpsisp2.24’) and ^1^H-^1^H TOCSY (‘mlevgpphw5’) experiments. The ^1^H-^13^C HSQC spectra were measured with acquisition times of 63.9 ms (^1^H) and 50.9 ms (^13^C), a 3 s pulse delay, 8 scans and acquisition of 2048 data points (^1^H) in 768 increments (^13^C). The ^1^H and ^13^C pulse widths were p1 = 7.44 μs and p3 = 9.3 μs, respectively. The ^1^H and ^13^C spectral widths were 20 ppm and 100.00 ppm, respectively. ^1^H-^1^H TOCSY spectra were acquired with the same proton pulse width as for the ^1^H-^13^C HSQC. The spectral widths were13.95 ppm in both dimensions, the acquisition times were 183.5 ms (F2) and 229 ms (F1), the ^1^H-^1^H TOCSY mixing times 80 ms and the pulse delay 2 s. 8 scans were used, 2048 points and 512 increments were acquired in the direct and indirect dimensions, respectively.

Sodium phosphate (Na_2_HPO_4_), imidazole, and sodium azide (NaN_3_) were bought from SigmaAldrich, deuterium oxide (D_2_O) from Cambridge Isotopes, and 3-(trimethylsilyl) propionic-2,2,3,3-d_4_ acid sodium salt (TSP-d_4_) from MerckMillipore.

#### Annotation of metabolites

1D proton, 2D ^1^H-^13^C HSQC, and 2D ^1^H-^1^H TOCSY spectra of pooled serum from all individuals in the dataset were used for metabolite identification. Chenomx NMR suite 8.1 (Chenomx Inc., Edmonton, Canada) was used for spectral line fitting of 1D proton spectra. Chemical shifts in 1D proton, 2D ^1^H-^13^C HSQC, and 2D ^1^H-^1^H TOCSY spectra were compared with reference spectra in the Human Metabolome Database (HMDB) [20].

### Data pre-processing and statistical analyses

#### Data pre-processing

^1^H-NMR spectra were aligned using icoshift and manual integration of peaks was performed to a linear baseline on all spectra in parallel using an in-house MatLab (MathWorks, Natick, USA) routine. In total 296 peaks were integrated within chemical shift range of 0.721–8.362 ppm. Data were normalized using Probabilistic Quotient Normalization (PQN).

#### Principal component analysis

Principal component analysis (PCA) was performed using SIMCA software v.14.1 (Umetrics AB, Umeå, Sweden) [21]. PCA models were used to explore clustering patterns of observations, trends in the data and outliers. Two samples were removed due to poor data quality. Samples from each breakfast group were modelled separately to identify outliers, resulting in exclusion of 2 of 96 for the CB samples and 6 of 96 for the EHB samples according to Hotellings T2 range (Tcrit 99%) and Distance to Model (DModX) (not exceeding 1.8). In total, 182 samples were included in further data analysis. Two variables were removed from the data set (imidazole (pH indicator)). In total, 294 variables were included in the model for further analysis.

#### Orthogonal projections to latent structures with discriminant analysis and orthogonal projections to latent structures with effect projections

Orthogonal Projections to Latent Structures with Discriminant Analysis (OPLS-DA) is a multivariate analysis tool that is often used in metabolomics [13]. However, OPLS-DA does not account for dependent data, such as repeated measures on the same individual which is generated in cross-over studies [22, 23]. OPLS-Effect Projections (EP) is a newly developed multivariate analysis that considers pairwise dependent samples in cross-over studies [15]. Both methods were performed in the present study using SIMCA software v.14.1 (Umetrics AB, Umeå, Sweden).

In the OPLS-DA and OPLS-EP models, four additional, highly abundant signals from unidentified lipids were removed since they, as a consequence of using Pareto scaling, influenced the model merely on account of the magnitude of the peaks. However, these lipids did not differ significantly between breakfast treatments (Mann Whitney U-test *p* > 0.05). Furthermore, the use of T2-filtered NMR experiment on water samples negated any identification of individual hydrophobic lipids and fatty acids. Hence, lipid variables did not contribute to the biological understanding of the data and OPLS models were not discernably affected by the removal of these variables. The numbers of latent variables (LV) in the models were determined using cross validation and Q^2^.

The validity of the OPLS models was assessed using Coefficient of Variation- ANalysis Of VAriance testing of cross-validated predictive residuals (CV-ANOVA), the cumulative amount of explained variation in the data summarized by the model (R^2^X[cum] and R^2^Y[cum]) and the predictive ability of the model (Q^2^[cum]). In addition, permutation tests (*n* = 999) was used for validation. For the OPLS-EP model, scores in relation to the response vector (Y) was also used for validation [15].

Separation of classes and variables related to separation in the data according to classification of breakfast meals was evaluated using OPLS-DA. Prior to modeling, data were centered and Pareto scaled. Cross validation groups were set to 24 (i.e. to number of study participants) and assigned observations based on observation ID for each individual so that all samples from one individual were left out in one cross-validation round. Receiver operating curve (ROC) analysis was performed and the area under the curve (AUC) was used as an estimate of the predictive accuracy of each breakfast meal in the OPLS-DA model. To select class discriminating variables of interest for annotation, S-plot, loadings (pq > 0.1) and top ranked variables in variable importance (VIP) scores in the OPLS-DA model were assessed.

For the OPLS-EP analysis, an effect matrix was calculated with Excel 2010. Mean values for each breakfast meal were calculated and values from the CB were subtracted from the EHB values for each volunteer (*n* = 24) and variable (*n* = 290). The effect matrix, i.e. the difference between breakfast meals, was modeled in relation to a response vector (Y = 1). Prior to modeling in Simca, all data were Pareto scaled (ParN) but not centered, and cross validation groups were set to 7 (default). S-plot was used for selection of variables of interest for annotation.

#### ANOVA-decomposed projections to latent structures

Multilevel methods, such as OPLS-EP, are capable of managing sample dependency in a cross-over design, but are in this case limited to comparing mean values between treatments. To simultaneously manage sample dependency and include all samples in the analysis, ANOVA decomposition [16] of the data was performed, by the factors: *Coffee/Tea*; *Gender*; *Individual*; and *Breakfast*. The factor *Size of breakfast* was almost completely confounded by *Gender*, wherefore it was excluded from ANOVA decomposition. Following the ANOVA-PLS approach [17], residuals were then added back to the breakfast type data, and analyzed by PLS to investigate systematic differences in the metabolome as a consequence of breakfast type. This supervised model was constructed in a repeated double cross validation procedure (rdCV) [24, 25], and incorporated with unbiased variable selection obtained by recursive feature elimination in the inner rdCV loop [26]. All samples per individual were co-sampled into the same cross validation segments to avoid overfitting to dependent samples. This approach has previously proven successful for supervised multivariate modelling [27, 28] to produce robust predictive modelling with effective variable selection and minimized risk of false positive discovery and model overfitting. Model performance was further assessed by permutation tests (*n* = 300) [29]. These analyses were performed in R v. 3.4.2 using in-house scripts, available from the authors upon request.

#### Significance tests of variables in multivariate models

Univariate statistical analysis of variables was performed using Mann Whitney U-test for the OPLS-DA model and Wilcoxon signed rank test for the OPLS-EP model; both with Benjamini Hochberg correction at the 95% level. A variable was considered significant if *p* < 0.05. These calculations were performed in MatLab R2015a.

## Results

### Breakfast related metabolic profiles

**Fig. 2:**
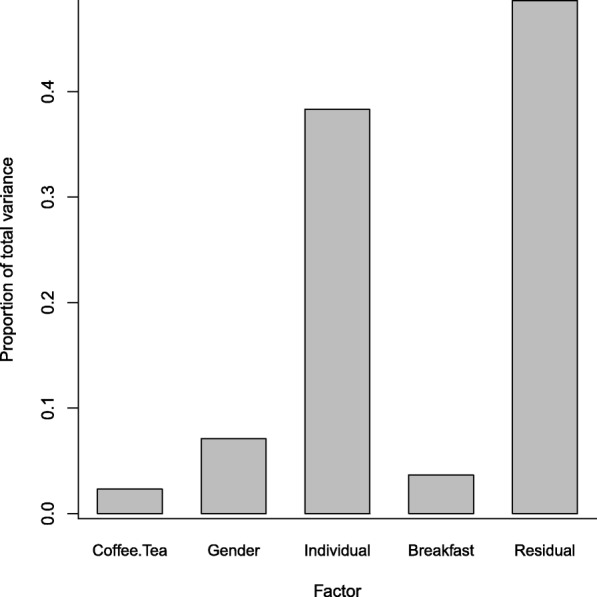
ANOVA decomposition visualising proportion of total variance in relation to different factors in postprandial (3 h) serum samples (*n* = 182) from 24 healthy volunteers

Statistics for the final OPLS models are presented in Table 2. Overall, the OPLS-DA model correctly classified 92% of the postprandial samples from the CB and 90% of the postprandial samples from the EHB while the ANOVA-PLS correctly classified 99% of samples with respect to breakfast type (p_permutation_ = 6.70e-17) (Table 3). ANOVA decomposition of the original data showed that the residuals represented the largest source of variability in the data (49%). Structured variability was dominated by inter-individual variation (38%) while breakfast intervention-related variability comprised 3.6% (Fig. 2).

**Table 2:** OPLS models statistics

Model	Nr of LV^a^	n	R^2^X [cum]^b^	R^2^Y [cum]^c^	Q^2^ [cum]^d^	CV-ANOVA^e^ (*p*-value)	Permutation test (Q^2^)^f^
OPLS-DA^g^	1 + 2 + 0	182	0.428	0.712	0.619	< 0.001	−0.165
OPLS-EP^h^	1 + 2 + 0	24	0.656	0.966	0.922	< 0.001	–

^a^Latent Variables

^b^ Cumulative fraction of the sum of squares of X explained by the selected latent variables

^c^ Cumulative fraction of the sum of squares of Y explained by the selected latent variables

^d^ Cumulative fraction of the sum of squares of Y predicted by the selected latent variables, estimated by cross validation

^e^ ANalysis Of VAriance testing of Cross-Validated predictive residuals

^f^ The intercept between real and random models, degree of overfit

^g^ Orthogonal Projections to Latent Structures with Discriminant Analysis

^h^ Orthogonal Projections to Latent Structures with Effect Projections

^–^ Not applicable

**Table 3:** Classification of postprandial serum samples by different models

True intake	Classification				
	OPLS-DA^a^		ANOVA-PLS^b^		
	CB	EHB	CB	EHB	*Total (n)*
CB^c^	85 (92%)	7 (8%)	91 (99%)	1 (1%)	92
EHB^d^	9 (10%)	81 (90%)	1 (1%)	89 (99%)	90
*Total*	94	88	92	90	182

^a^Orthogonal Projections to Latent Structures with Discriminant Analysis (Cross-validated scores)

^b^ANalysis Of Variance - Partial Least Squares

^c^Cereal breakfast

^d^Egg and ham breakfast

**Fig. 3:**
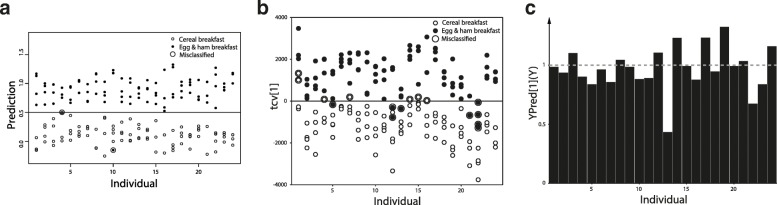
**a** Predicted values for breakfast classification in ANOVA-Partial Least Squares (PLS) model. The data were ANOVA-decomposed into the factors Coffee/Tea, Gender, Individual and Breakfast type and PLS analysis was performed on the Breakfast type data after addition of the residual matrix. Model included 290 variables and 182 postprandial observations from 24 healthy volunteers. **b** Breakfast dependent cross-validated scores (cross-validated x scores (tcv)) in orthogonal projections to latent structures with discriminant analysis (OPLS-DA) model. Model included 290 variables and postprandial (3 h) serum samples (*n* = 182) from 24 healthy volunteers. **c** Predicted values in relation to response vector (Y) for volunteers in the in OPLS with effect projections (EP) model. The dotted line (Y = 1) indicates the response vector value for the model. The magnitude of the predicted effect for each volunteer is given by the height of the corresponding black bar. Deviations from the value 1 for a specific volunteer indicate a larger (> 1) or smaller (< 1) metabolic effect (difference between breakfast meals) in the model direction (metabolic profile) associated with the metabolism of foods included in the different breakfast meals. Model included 24 observations (equal to number of individuals) and 290 variables

**Fig. 4:**
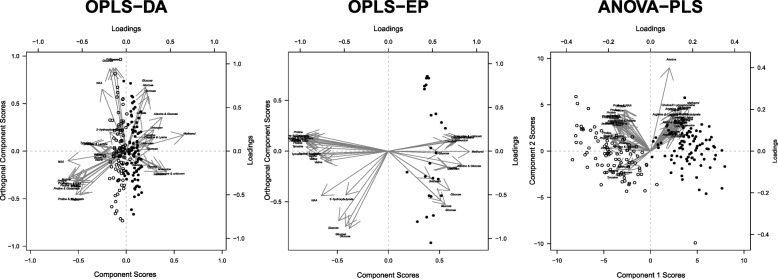
Biplot in Orthogonal Projections to Latent Structures with Discriminant Analysis (OPLS-DA), OPLS with Effect Matrix (EP) and ANOVA-Partial Least Squares (PLS) models of observation scores and variable loadings. OPLS-DA and ANOVA-PLS models included 182 postprandial observations from 24 healthy volunteers and 290 variables while the OPLS-EP model 24 observations (equal to number of individuals) and 290 variables. Labeled metabolites denote selected discriminating metabolites in the different models

**Fig. 5:**
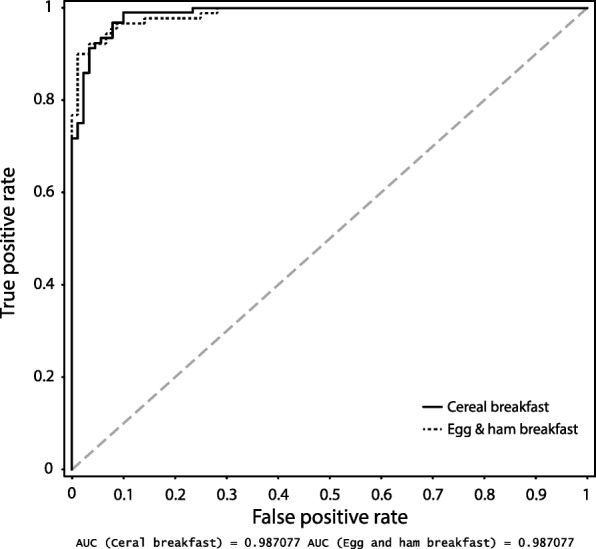
ROC curve in orthogonal projections to latent structures with discriminant analysis (OPLS-DA) model comparing the postprandial metabolic response between cereal breakfast (CB) and egg & ham breakfast (EHB). In total 182 serum samples from 24 individuals were included in the model, 90 samples from volunteers who had consumed the EHB and 92 samples from volunteers who had consumed the CB. On average four samples per individual and breakfast meal. AUC = area under the curve

The ability of the OPLS-DA, OPLS-EP, and ANOVA-PLS models to discriminate between metabolic profiles of postprandial samples from volunteers who had consumed the CB and the EHB are displayed in Figs. 3 and 4. Seven out of 92 postprandial samples from the CB and nine out of 90 postprandial samples from the EHB were misclassified in the OPLS-DA model. However, when looking at the OPLS-DA model predictions (Fig. 3b), only two individuals have consistent erroneous breakfast predictions. The predictive accuracy for each breakfast meal by the OPLS-DA model, as illustrated by the area under the curve in ROC analysis, is displayed in Fig. 5. Metabolites discriminating in the OPLS-DA model were selected using a combination of S-plot (Additional file 4), loadings, and top ranked variables in VIP scores in the OPLS-DA model.

In the OPLS-EP model, all values above 0 in the OPLS-EP model correctly predicted the order of consumption of the EHB or the CB, whereas a negative value would predict the opposite. Only one individual displayed < 0.5 in predictive effect in relation to the response vector (Y), whereas remaining volunteers displayed > 0.75 in predictive effect (Fig. 3c). Again, metabolites increasing or decreasing in relation to the response vector were selected using S-plot (Additional file 5).

Further, the influence of a single meal on the postprandial metabolic profile the following day was studied by the difference in metabolic profiles between the same breakfast meal in relation to the breakfast meal served the day before. However, OPLS-DA models displayed low prediction (Q^2^) (data not shown) in class separation between breakfast meals, consistent with the ANOVA-PLS analysis showing no importance of the order of breakfast meals.

### Discriminating metabolites

In total, seven unique metabolites were identified that were selected responsible for class separation in all models. Tyrosine, proline, and N-acetylated amino acid were found in significantly higher concentrations after consumption of the CB. In contrast, alanine, methanol, creatine, and isoleucine were found in significantly higher concentrations after consumption of the EHB.

For the OPLS models, five and six (excluding unknowns and glucose) metabolites were identified as discriminating in postprandial samples from the CB and the EHB respectively (Table 4). The ANOVA-PLS model unbiasedly selected two components and 48 variables corresponding to 12 metabolites as top predictors driving the separation between classes with three and nine metabolites responsible for class separation in postprandial samples from the CB and the EHB respectively. 3-hydroxybutyrate, valine, and glycine were selected as responsible for class separation in OPLS models but not in ANOVA models. In contrast, arginine, lysine, 4-aminobutyrate, choline, and glutamine were selected as top ranked variables in ANOVA-PLS but not in the OPLS models. All identifications of metabolites, except 3-hydroxybutyrate, were supported by 2D ^1^H-^13^C HSQC data.

**Table 4:** Discriminating metabolites between breakfast meals in multivariate models

Meal	Metabolite	Chemical shift (ppm)	*p*-value^a^	Model
CB^b^	Tyrosine	7.19^c^, 6.89	< 0.0001	All^d^
CB	Proline	**4.13**^e^, 4.14, **4.15**, 3.33, **3.34**, **3.35**, **2.37**, 2.36, 2.35, **2.34**, **2.33**, **2.10**, **2.09**, *2.07*^f^, 2.06, **2.03**, 2.02, 2.01, 2.00, 1.99, **1.98**, **1.97**	< 0.0001	All
CB	NAA^g^	**2.09**, 2.08, 2.06, *2.05*, **2.03**, **1.99**	< 0.0001	All
CB	3-hydroxybutyrate	*1.20*	0.014	OPLS-DA^h^, OPLS-EP^i^
CB	Valine	*1.05* *, 1.04, 1.00, 0.99*	0.0004	OPLS-DA, OPLS-EP
CB	(Glucose)^j^	*5.24* *, 3.91, 3.89*	0.005	OPLS-DA, OPLS-EP
CB	Unknown	**3.44**, **1.15**, **1.14**	–	ANOVA-PLS^k^
EHB^l^	Methanol	3.37	< 0.0001	All
EHB	Creatine	3.94, 3.04	< 0.0001	All
EHB	Isoleucine	1.97, 1.98, 1.02, 1.01, 0.95, 0.94, 0.93	< 0.0001	All
EHB	(Alanine)^m^	3.79	< 0.0001	All
EHB	Arginine	1.89, 1.90, 1.91, 1.92	< 0.0001	ANOVA-PLS
EHB	Lysine	**3.03**, **3.02**, **1.90**, **1.91**, **1.92**	< 0.0001	ANOVA-PLS
EHB	4-aminobutyrate	**1.90**	< 0.0001	ANOVA-PLS
EHB	Choline	**3.21**	< 0.0001	ANOVA-PLS
EHB	Glutamine	**2.16**	< 0.0001	ANOVA-PLS
EHB	(Glucose)^j^	*3.86, 3.74,* *3.72* *, 3.49*	0.0006	OPLS-DA, OPLS-EP
EHB	Lactate	*4.11*	0.60	OPLS-DA
EHB	Glycine	*3.57*	0.0002	OPLS-DA, OPLS-EP
EHB	Unknown	**4.05**, *3.77*, *3.73,* **3.68**, **2.56**	–	All

^a^Wilcoxon signed rank test

^b^Cereal breakfast

^c^Underscored chemical shift used for *p*-value calculation and variable rank number

^d^Includes ANOVA-PLS, OPLS-DA, and OPLS-EP models

^e^Bold chemical shifts indicate discriminating variable only in ANOVA-PLS model

^f^Italic chemical shifts indicate discriminating variable only in OPLS-DA and OPLS-EP models

^g^N-acetylated-amino acids

^h^Orthogonal Projections to Latent Structures with Discriminant Analysis

^i^Orthogonal Projections to Latent Structures with Effect Projections

^j^Unknown masked by glucose

^k^ANalysis Of Variance – Partial Least Squares

^l^Egg and ham breakfast

^m^Overlap with glucose and unknown metabolites

– Not relevant

## Discussion

Evaluating the outcome of three different multivariate statistical approaches, OPLS-DA, OPLS-EP and ANOVA-PLS, in a cross-over design, including 24 healthy volunteers, we explored the postprandial metabolic response to two isocaloric breakfast meals with similar macronutrient distribution, but with different food items.

### Multivariate statistical modelling of metabolic profiles

The OPLS-DA model correctly classified of 92 and 90% of serum metabolic profiles the CB and the EHB meals, respectively (Table 3 and Fig. 3b). Moreover, the OPLS-DA model showed an overall difference in metabolic profiles between breakfast meals with a high degree of intra-individual similarity, although inter-individual variability was clearly discernible (Fig. 4). Similar to these findings, Lenz et al. (2003) [30] found relatively low inter- and intra-individual variation in ^1^H-NMR plasma metabolic profiles when providing a semi standardized diet during 2 days to healthy males. Likely, the confounding effect of inter-individual variability depends on the effect size of the intervention.

OPLS-EP takes pairwise sample dependency into account by modelling the effect matrix rather than the original data [15]. When better accounting for the paired structure of the data using OPLS-EP, the predictive ability of the model improved compared to the OPLS-DA model (Table 2). This confirms the confounding effect of inter-individual variability (Fig. 4). In addition, the OPLS-EP model classified all individuals correctly using 7-fold cross validation.

ANOVA-PLS was developed to allow for supervised PLS analysis of data decomposed by one or several factors, which makes it efficient to manage e.g. cross-over dependency by including *Individual* as a factor [17]. Although not shown here, it can be shown that multilevel approaches are special cases of ANOVA decomposition; the latter being the broader framework. In fact, their relation is conceptually similar to the difference between a paired t-test and a classical ANOVA. The ANOVA decomposition step thus manages not only to isolate inter-individual variation, but also to isolate other factors that may otherwise confound the analysis (in this case *Coffee/Tea* consumption and *Gender*), analogously to how such factors may be included in linear mixed models to account for confounding variables [16]. This approach provides a means to investigate contributions of the factors to the total variance by comparing sums of squares. In our data, the factor *Individual* was by far the major contributor to systematic variability, although overshadowed by the residual variability. This clearly indicates between sample fluctuations as the major source of variability, although the source of such fluctuations was not investigated. It is likely, however, that such variability is composed of both biological variation in the individual, pre-analytical sample management and instrumental variability [31]. It should be noted that the sums-of-squares in the currently used function were calculated sequentially (i.e. Type I sums-of-squares) and are thus sensitive to the order of factors if the design is not balanced. Sensitivity analysis, however, revealed only minor effect of the order of factors on both sums-of-squares and modelling outcomes in the present case.

Similar to OPLS-EP, classes were visibly separated in the ANOVA-PLS (Fig. 4). ANOVA-PLS thus managed to improve classification accuracy compared to OPLS-DA, even though this procedure employed a much stricter validation scheme than either of the OPLS methods [24]. This clearly shows the advantages of filtering out inter-individual variability prior to analysis to be able to focus on systematic differences between treatments. Using this approach, all samples were maintained in the analysis, leading to higher resolution in the multivariate model compared to OPLS-EP (Fig. 4). This also provided an opportunity to investigate whether effects were robust even with residual variability from multiple samples. ANOVA-PLS thus effectively combined the best aspects of discriminant and standard multilevel (such as Effect Projections) analyses for analysing complex cross-over data structures. Permutation analysis showed that the ANOVA-PLS was highly significant and devoid of overfitting (Additional file 6), since the permutation distribution median corresponded exactly to the expected value of 91 misclassifications for 182 randomly permuted observations in a two-class problem.

The order of individual breakfast meals did not have an impact on the metabolic profile in the present study, implying that foods in our breakfast meals do not influence the postprandial serum metabolic profiles measured by NMR after 24 h. Previously, the fat to carbohydrate ratio of an evening meal has been shown to impact the postprandial metabolic response in plasma 12 h after intake [32]. However, in our study the breakfast meals contained the same fat to carbohydrate ratio and this might explain why the postprandial response did not seem to be influenced by the type of breakfast consumed the day before. Moreover, white bean consumption has been shown to affect metabolite concentrations in urine up to 48 h after intake in a previous study [33]. However, the effect was not captured in serum, and this is in line with findings in the present study.

### Discriminating metabolites

The cross-over design has the advantage of comparing each individual to themselves after the two different breakfasts. Given the use of proper statistical tools factors such as age [34], gender [35], BMI [36], insulin sensitivity [37], habitual diet [38], and habitual sleep [39], have minimal impact on the results. Although inter-personal variability confounded dietary effects in OPLS-DA, model predictions and biplots from all statistical methods provided similar output (Figs. 3 and 4). This was confirmed by the fact that discriminating metabolites selected from both OPLS models were, in fact, the same (Fig. 4 and Table 4). The difference in the ANOVA-PLS model is likely to a large extent dependent on the different procedures for variable selection. However, several variables were selected in all models (Fig. 4 and Table 4), suggesting that these may be the most robust findings.

The metabolites that were discriminating in all statistical analysis are thus discussed first. Variables including glucose, alanine, and lactate that discriminated between breakfasts in OPLS models had peaks overlapping with other metabolites or were not significant in univariate models and, since their relevance is therefore unclear, these metabolites are not discussed further.

Proline and tyrosine were found at higher concentrations in postprandial serum samples from the CB in relation to the EHB. Consistently, the CB had a higher proline content, with the major contribution from the cheese (Additional files 1 and 2). In contrast, there was little difference in total tyrosine content between the two breakfast meals, with sources as hard cheese in the CB and white beans and pork loin in the EHB. Tyrosine has previously been reported higher in postprandial serum samples, following a dairy meal compared to similar tyrosine content from fish, meat and lentils [40]. In our study the higher tyrosine in the CB may be related to the absorption and ratio between free and bound amino acids and/or the metabolic turnover of the different foods within the breakfasts [41].

The higher concentration of *N*-acetylated amino acids (NAA) in the CB compared to the EHB, might be related to a higher beta-oxidation rate of fatty acids, since the CB had a higher content of short chain fatty acids (Additional file 3) [42]. NAAs and N-acetylcysteine in particular, has shown various biological activities such as interaction with pathways regulating cell cycle and apoptosis, immune-modulation and gene expression among others [43, 44]. Unfortunately, however, it was not possible to unequivocally identify involved N-acetylated amino acids in this study.

For the EHB, methanol, creatine and isoleucine were higher than for the CB. The metabolism of fruit and vegetables, mainly due to the pectin content that is metabolized by the gut microflora, is believed to generate the majority of the serum methanol originating from the diet, other than alcoholic beverages [45, 46]. Together with the tomatoes and white beans, the orange marmalade, high in natural as well as added pectin, likely caused the higher methanol concentration after the EHB. In similarity with previous findings creatine was found in higher concentration in postprandial samples where volunteer had consumed red meat [47]. Leucine, as well as other branched amino acids has previously been shown to be associated with dietary intake of animal products and pulses [48] and the serum concentration of isoleucine likely reflects the higher intake in these products in the EHB.

In the OPLS models valine, 3-hydroxybutyrate, and glycine were selected as discriminating metabolites and these also differed between breakfast groups. Higher serum glycine was found after consuming the EHB that included both legumes and red meat, which are both major dietary sources of glycine. Glycine is a non-chiral amino acid that has also been identified as an endogenous metabolite in plasma [49]. Further, the EHB had a higher content of valine compared to the CB. Nevertheless, postprandial samples from the CB had higher concentrations of valine. The main food that contributed to the valine content in the CB was the hard cheese, whereas in the EHB it was white beans in tomato sauce. Our results suggest that absorption rates of valine differ between these foods. The ketone 3-hydroxybutyrate was higher after consumption of the CB. 3-hydroxybutyrate has previously been related to fat: carbohydrate ratio [32], but since macronutrients were balanced in the two breakfast meals we speculate that the observed difference in serum may be related to the composition of the fatty acid profiles of the meals (Additional file 3).

The ANOVA-PLS model also included arginine, lysine, glutamine, choline and, 4-aminobutyrate as discriminating metabolites. This reflected the fact that the EHB had higher content of all amino acids, except proline, tyrosine and tryptophan (Additional file 1), with white beans (arginine, lysine and, glutamine) and animal product as dietary sources. Animal products also constitute rich sources of choline [50] which might explain the higher serum concentration in volunteers who had consumed the EHB.

### Strengths and limitations

Limitations of this study include that the study design did not allow us to investigate specificity of metabolic responses to the included foods, which is necessary for biomarker discovery and validation, or long-term metabolic effects. To evaluate potential health implications and/or biomarker applications of specific food items, other study designs are needed. However, the aim of this study was to evaluate the acute metabolic serum response between two complex meals. Further, specific N-acetylated amino acids, metabolites with low abundance in overlapping regions and individual fatty acids could not be identified. This inability is related to the NMR analysis of the current sample matrix. In addition, the included volunteers in the present study constituted a fairly homogenous group of individuals which is considered a limitation for the generalizability of the results. A larger and more heterogeneous group of volunteers may be preferable to reflect the metabolic response in the general population. Strengths of the study include the cross-over design to be able to focus on systematic intra-individual effects of dietary interventions, the standardized prior evening meal, the equal numbers of women and men, the repeated sampling which gave the opportunity to investigate sample-by-sample variability in relation to systematic dietary effects and, the application of several statistical tools.

## Conclusions

In conclusion, all statistical models successfully separated metabolic profiles between the two breakfast meals, but with some limitations: OPLS-DA could not effectively manage sample dependency from the cross-over design, OPLS-EP could not manage repeated sampling per treatment, whereas ANOVA-PLS effectively managed both the cross-over and repeated measures design. When having dependent samples, OPLS-EP and ANOVA-PLS thus have the means to handle the data structure and generate more robust models with higher predictive performance than OPLS-DA. It is thus necessary that multivariate models considering sample dependency should be applied in cross-over metabolomics studies. Using NMR-metabolomics, especially when combined with appropriate multivariate models, it was possible to identify difference in serum metabolic profiles between two isocaloric meals with the same macronutrient composition yet including different foods. The differences in metabolites, discriminating between breakfast meals, largely mirrored differences in dietary composition. Thus, metabolomics holds the potential to complement traditional methods to evaluate dietary intake and compliance in intervention studies.

## Additional files

Additional file 1: **Table S1.** Amino acid content of breakfast meals (mg). (DOCX 92 kb)

Additional file 2: **Table S2.** Amino acid content of individual foods (mg). (DOCX 94 kb)

Additional file 3: **Table S3.** Fatty acid content of breakfast meals (g). (DOCX 92 kb)

Additional file 4: S-plot in orthogonal projections to latent structures with discriminant analysis (OPLS-DA) model comparing the postprandial metabolic response between cereal breakfast (CB) and egg & ham breakfast (EHB). The top box is displaying selected discriminating metabolites for the EHB while the bottom box is displaying selected discriminating metabolites for the CB. Grey circles indicate significant (*p* < 0.05) variables in Mann Whitney U-test. (EPS 5517 kb)

Additional file 5: S-plot in orthogonal projections to latent structures with effect matrix (OPLS-EP) model comparing variables increasing and decreasing in relation to the response vector (Y). The top box is displaying selected metabolites increasing and the bottom box is displaying metabolites decreasing in relation to Y. Grey circles indicate significant (*p* < 0.05) variables in Wilcoxon signed rank test. (EPS 890 kb)

Additional file 6: Permutation analysis of ANOVA-PLS, showing actual model misclassifications as vertical lines with *p*-value calculated as the cumulative probability of finding the actual model result is a Student’s t-distribution of misclassification results obtained from randomly permuted data (*n* = 300 per model). The permutation distribution had median value of 91 misclassifications, corresponding exactly to the expected value of 182 observations in a two-class problem. The results show strong predictive modelling performance with absence of overfitting in the validation frameworks. (EPS 418 kb)

## Abbreviations

ANOVA: ANalysis Of Variance
BMI: Body Mass Index
CB: Cereal Breakfast
CV-ANOVA: Coefficient of Variation- ANalysis Of Variance
EHB: Egg & Ham Breakfast
HMDB: Human Metabolome Database
HSQC: Heteronuclear Single-Quantum Correlation
MET: Metabolic Equivalent
NAA: N-acetylated amino acids
NMR: Nuclear Magnetic Resonance
NOESY: Nuclear Overhauser Effect SpectroscopY
OPLS-DA: Orthogonal Projections to Latent Structures with Discriminant Analysis
OPLS-EP: Orthogonal Projections to Latent Structures with Effect Projections
PCA: Principal Component Analysis
PLS: Projektions to Latent Structures
rdCV: Repeated double Cross Validation
ROC: Receiver Operating Curve
TOCSY: Total Correlation SpectroscopY
