# Supplementary material for: The 1H NMR serum metabolomics response to a two meal challenge: a cross-over dietary intervention study in healthy human volunteers
Source: Nutr J. 2019 Apr 8;18:25. doi: 10.1186/s12937-019-0446-2 (PMC6454665; doi:10.1186/s12937-019-0446-2)
Supplement: Supplementary file 1 — Table S1. Amino acid content of breakfast meals (mg). (DOCX 92 kb) [file 12937_2019_446_MOESM1_ESM.docx]

| **Table S1. Amino acid content of breakfast meals (mg)** | | | | |
| --- | --- | --- | --- | --- |
|  | Cereal Breakfast |  | Egg & Ham Breakfast | |
| Amino acid | 500 kcal | 750 kcal | 500 kcal | 750 kcal |
| Isoleucine | 1054 | 1485 | 1258 | 1943 |
| Leucine | 1720 | 2476 | 2074 | 3235 |
| Lysine | 1459 | 2032 | 1900 | 2848 |
| Methionine | 486 | 705 | 561 | 851 |
| Cysteine | 203 | 300 | 315 | 508 |
| Phenylalanine | 1020 | 1479 | 1271 | 2024 |
| Tyrosine | 824 | 1224 | 846 | 1299 |
| Threonine | 721 | 1025 | 1081 | 1669 |
| Tryptophan | 259 | 375 | 304 | 474 |
| Valine | 1352 | 1941 | 1510 | 2360 |
| Arginine | 923 | 1288 | 1629 | 2474 |
| Histidine | 550 | 790 | 754 | 1142 |
| Alanine | 760 | 1091 | 1287 | 1943 |
| Asparagine | 1649 | 2273 | 2834 | 4326 |
| Glutamine | 4097 | 5929 | 4341 | 6787 |
| Glycine | 587 | 874 | 1040 | 1597 |
| Proline | 2060 | 2909 | 1464 | 2215 |
| Serine | 1113 | 1586 | 1401 | 2215 |
