# Supplementary material for: The 1H NMR serum metabolomics response to a two meal challenge: a cross-over dietary intervention study in healthy human volunteers
Source: Nutr J. 2019 Apr 8;18:25. doi: 10.1186/s12937-019-0446-2 (PMC6454665; doi:10.1186/s12937-019-0446-2)
Supplement: Supplementary file 2 — Table S2. Amino acid content of individual foods (mg). (DOCX 94 kb) [file 12937_2019_446_MOESM2_ESM.docx]

| **Table S2. Amino acid content of individual foods (mg)** | | | | | | | | | | | | |
| --- | --- | --- | --- | --- | --- | --- | --- | --- | --- | --- | --- | --- |
|  | Cereal Breakfast (750 kcal) | | | | | Egg & Ham Breakfast (750 kcal) | | | | | | |
| Amino acid | Milk (3%) | Oat puffs | Rye bread | Hard cheese (28%) | Tomatoe | Milk (3%) | Lightly smoked pork loin | White beans in tomato sauce | Egg | Tomato | Orange marmalade | White bread |
| Isoleucine | 441 | 199,5 | 151,2 | 652,5 | 2,88 | 73,5 | 475,2 | 763,2 | 379,6 | 25,2 | 3,6 | 185 |
| Leucine | 651 | 364 | 248,4 | 1147,5 | 4,56 | 108,5 | 744 | 1363,2 | 598 | 39,9 | 5,76 | 315 |
| Lysine | 630 | 210 | 145,8 | 967,5 | 4,56 | 105 | 835,2 | 1172 | 514,8 | 39,9 | 7,2 | 100 |
| Methionine | 189 | 80,5 | 64,8 | 360 | 0,24 | 31,5 | 254,4 | 256 | 228,8 | 2,1 | 1,08 | 67,5 |
| Cysteine | 52,5 | 136,5 | 59,4 | 43,65 | 1,68 | 8,75 | 77,28 | 185,6 | 135,2 | 14,7 | 0,72 | 80 |
| Phenylalanine | 357 | 266 | 194,4 | 616,5 | 3,12 | 59,5 | 374,4 | 927,2 | 369,2 | 27,3 | 3,96 | 220 |
| Tyrosine | 273 | 154 | 72,9 | 697,5 | 2,16 | 45,5 | 326,4 | 490,4 | 291,2 | 18,9 | 2,52 | 100 |
| Threonine | 273 | 168 | 124,2 | 418,5 | 3,12 | 45,5 | 403,2 | 708,8 | 317,2 | 27,3 | 3,6 | 125 |
| Tryptophan | 90,3 | 66,5 | 43,2 | 162 | 0,96 | 15,05 | 98,4 | 201,6 | 93,6 | 8,4 | 1,08 | 44 |
| Valine | 504 | 297,5 | 221,4 | 855 | 3,12 | 84 | 504 | 900 | 494 | 27,3 | 5,76 | 285 |
| Arginine | 252 | 322 | 183,6 | 369 | 3,12 | 42 | 566,4 | 1063,2 | 431,6 | 27,3 | 15,12 | 170 |
| Histidine | 205,8 | 112 | 86,4 | 360 | 1,8 | 34,3 | 340,8 | 463,2 | 166,4 | 15,75 | 2,52 | 95 |
| Alanine | 252 | 248,5 | 167,4 | 369 | 3,48 | 42 | 566,4 | 708,8 | 400,4 | 30,45 | 5,04 | 140 |
| Asparagine | 567 | 392 | 280,8 | 796,5 | 16,8 | 94,5 | 864 | 2072 | 702 | 147 | 20,88 | 205 |
| Glutamine | 1470 | 1050 | 972 | 2272,5 | 45,6 | 245 | 1272 | 2590,4 | 800,8 | 399 | 11,16 | 1350 |
| Glycine | 138,6 | 273 | 178,2 | 238,5 | 3,12 | 23,1 | 432 | 654,4 | 239,2 | 27,3 | 3,96 | 175 |
| Proline | 693 | 266 | 405 | 1282,5 | 2,28 | 115,5 | 340,8 | 708,8 | 265,2 | 19,95 | 24,84 | 480 |
| Serine | 399 | 273 | 199,8 | 634,5 | 3,24 | 66,5 | 374,4 | 927,2 | 514,8 | 28,35 | 7,56 | 220 |
