# Supplementary material for: The 1H NMR serum metabolomics response to a two meal challenge: a cross-over dietary intervention study in healthy human volunteers
Source: Nutr J. 2019 Apr 8;18:25. doi: 10.1186/s12937-019-0446-2 (PMC6454665; doi:10.1186/s12937-019-0446-2)
Supplement: Supplementary file 3 — Table S3. Fatty acid content of breakfast meals (g). (DOCX 92 kb) [file 12937_2019_446_MOESM3_ESM.docx]

| **Table S3. Fatty acid content of breakfast meals (g)** | | | | |
| --- | --- | --- | --- | --- |
|  | Cereal breakfast | | Egg and ham breakfast | |
| Fatty acid (g) | 500 kcal | 750 kcal | 500 kcal | 750 kcal |
| Saturated | 10,6 | 17,9 | 7,8 | 13,8 |
| 4:0-10:0 | 1,4 | 2,5 | 0,8 | 1,5 |
| 12:0 | 0,5 | 0,9 | 0,3 | 0,6 |
| 14:0 | 1,6 | 2,7 | 1 | 1,8 |
| 16:0 | 5 | 8,4 | 4 | 6,9 |
| 18:0 | 1,7 | 2,9 | 1,3 | 2,3 |
| Monounsaturated | 5,2 | 9,7 | 6,9 | 12,3 |
| 16:1 | 0,2 | 0,4 | 0,3 | 0,5 |
| 18:1 | 4,7 | 8,9 | 6,4 | 11,3 |
| Polyunsaturated | 1,3 | 2,6 | 2,6 | 4,6 |
| 18:2 | 1,1 | 2,1 | 1,7 | 3,1 |
| 18:3 | 0,3 | 0,6 | 0,7 | 1,3 |
| 20:4 | 0 | 0 | 0,1 | 0,1 |
| 22:6 | 0 | 0 | 0 | 0,1 |
